# State Tuberculosis Sanitorium at Carlsbad Has Bureau of Correspondence and Information

**Published:** 1918-12

**Authors:** 


### State Tuberculosis Sanatorium at Carlsbad Has Bureau of Correspondence and Information.

PURPOSE.

For the benefit of those who have tuberculosis, and for agencies interested in the stamping out of the disease the State Tuberculosis Sanatorium, located at Carlsbad, Tom Green County, Texas, maintains a Bureau of Correspondence and Information, for the dissemination of knowledge on the prevention and further spread of tuberculosis. The control of tuberculosis, and if possible, its elimination, is now considered to be of prime necessity to the public health of the people of Texas. The matter is primarily one of education and, recognizing this, the Thirty-fifth Legislature made an appropriation of $20,000.00 to be used by the Sanatorium, "For lecturing in colleges, schools and public gatherings, publishing pamphlets, books, and literature to be circulated including general work to educate the public, and prevent as much as possible the spread of tuberculosis."

SERVICE TO THE TUBERCULOUS.

The Bureau obtains names of the tuberculous from physicians, anti-tuberculosis societies, ex-patients of the Sanatorium and others. To the tuberculous, and others interested, are mailed pamphlets bearing on the treatment and prevention of tuberculosis. This matter is not uninteresting reading, and it would be a blessing to the State if every school teacher, every minister, every public speaker, and the head of every family in the State would read it. A special effort has been made to get in contact with Texas soldiers discharged from army camps on account of tuberculosis. These are supplied with literature and application blanks so that they may apply for entrance to the Sanatorium, if they so desire.

SERVICE TO THE PHYSICIAN

A majority of the physicians have expressed a desire to be furnished with reprints of articles by specialists on the early discovery of tuberculosis, readily admitting that they are deficient as to the methods of making early diagnosis. Once each month a carefully selected article on methods and treatment is mailed out to all the physicians of the State and it is believed this service is appreciated. Tuberculosis is one of the most curable of diseases, provided treatment is begun in time. If this was not true half of the population or more would die from it, because post-mortems tend to show that practically everyone has, at one time or another, an infection of tuberculosis.

IMPORTANCE OF INSTRUCTING CHILDREN.

It has been said that if all children could be protected from infection tuberculosis would be stamped out in a decade. This somewhat exaggerates the truth, but it is now believed by competent authorities that tuberculosis is rarely, if ever, contracted in adult life. The infection takes place in childhood and later, usually in young manhood at a time when the system is weakened either through disease, excesses, mental or physical strain, the breadkdown comes. It follows then; if children could be taught to avoid infection, or if their mothers and teachers could be taught to protect them from infection, a great deal would have been done towards doing away with the disease.

Fortunately, it is not a difficult matter to protect the young. To do so requires only that those who have active cases of tuberculosis be required to avoid infecting others. Practically the only way of spreading infection is through the sputum, therefore if the sputum of consumption was carefully collected and destroyed, one of the main sources would have been controlled. It is an easy matter for one to spit in paper sputum cups and later, burn the cups. Spitting at all is an unnecessary and disgusting habit but for a consumptive to spit around promiscuously is criminal. Those consumptives who fail to protect the public in this way should be placed in special institutions just as are lunatics or criminals. Other common ways of infecting others are: through kissing, and coughing and sneezing without covering the face. Kissing by consumptives should be prohibited, and spread of infection by coughing or sneezing prevented by covering the face with a paper napkin or muslin cloth, this napkin or cloth later to be burned. Where children are exposed to infection in the home the consumptive should be isolated from them as much as possible. Separate utensils should be provided—or at any rate all dishes used by the consumptive should be boiled after use.

NO DANGER TO OTHERS.

A careful consumptive is not a danger to anyone and if this fact could be borne in mind much mental anguish, much persecution of those who can least stand it would be avoided. This unreasoning fear of the consumptive is known as phthisiphobia. Quite often it proves to be the case that the consumptive who realizes his condition and takes proper precautions to protect others is subjected to annoyance, or ostracized, whereas a careless consumptive or one ignorant of his condition, without knowledge of protecting others, is permitted to mingle with well people and pursue his usual course of life, merely because he has never been "labeled."

SERVICE OF THE BUREAU OPEN TO ALL ALIKE.

If, as has been estimated, the economic loss to the State from tuberculosis is ninety million dollars per year, this alone should prove a cogent reason for active steps being taken to control the disease. However, the monetary loss is the least consideration in a proposition of this kind, because human lives and human happiness cannot be estimated in dollars and cents. With five thousand deaths per annum and more than fifty thousand lives blasted and ruined—an idea of the ravages of the disease is given. It was for the purpose of arousing the people of the State to action, so that a more active combatting of the disease might be entered into, that the Bureau was established. There was no thought of supplanting or hindering well established agencies and the Bureau's service is open to all; to physicians, anti-tuberculosis societies and others, and particularly to the tuberculous. One of the main functions of the Bureau will be to encourage the establishing of county sanatoria and preventoria, to furnish sputum cups and supplies to the tuberculous at cost, and to give information on the subject of tuberculosis, its prevention and treatment, to anyone who will receive it. To obtain such service address R. E. Luhn, Jr., Director, Bureau of Correspondence and Information, State Sanatorium, Carlsbad, Texas.

---

Do you want the editor of the Texas Medical Journal to have a joyous Christmas? Of course you do. Then, sit right down and send her your check for your subscription to the Journal, so it may reach her before Christmas day.

---

The *Medical Review of Reviews* announces that it has just purchased the third oldest medical journal in America—the Buffalo Medical Journal—founded seventy-four years ago by Dr. Austin Flint, and published regularly ever since.

